# cis-positional information in regulatory single nucleotide variation prioritization

**DOI:** 10.1093/bib/bbaf631.053

**Published:** 2025-12-12

**Authors:** Ka-Chun Wong, Zhongyu Yao, Weidun Xie, Zhongshen Li, Tianchi Lu, Cho Ling

**Affiliations:** Department of Computer Science, City University of Hong Kong, Hong Kong SAR; Department of Computer Science, City University of Hong Kong, Hong Kong SAR; Department of Computer Science, City University of Hong Kong, Hong Kong SAR; Department of Computer Science, City University of Hong Kong, Hong Kong SAR; Department of Computer Science, City University of Hong Kong, Hong Kong SAR; Department of Computer Science, City University of Hong Kong, Hong Kong SAR

## Abstract

Duttke et al. have proved and generalized past observations on the positional preferences of regulatory genomics at multiple functional levels in July 2024 [1]. However, the explicit open-box distribution learning on those positional preferences are under-explored in the existing gene regulation methods including deep learning. Contributing towards such directions, we propose to develop regulatory positional distribution models. The positional distribution models can capture the spatial features of gene transcription which can improve different downstream applications such as rSNV prioritization. Experiments have been conducted to substantiate its claim in deleterious rSNV predictions of ClinVar. In particular, we have collected the ClinVar dataset (i.e., ‘variant_summary.txt.gz’ on 2024-09-18) and retrieved all deleterious SNVs (i.e., labelled as ‘Pathogenic’ and ‘Likely pathogenic’ in the ‘ClinicalSigificance’ column) around all human TSS locations from Ensembl (i.e., Ensembl Genes 112). In particular, it was surprising that, although CADD is already an ensemble approach built upon different state-of-the-arts methods [2], CADD can still be improved with statistical significance after cis-positional information has been incorporated across different situations where evolutionary conservation signals (PhastCons and PhyloP) have been integrated. Based on the results, we propose two future research directions. The first direction is to examine different statistical distributions for position-aware gene regulation modelling while the second direction is to incorporate those distributions into different downstream applications such as eQTL analysis and deleterious rSNV predictions. The outcomes will have broad implications across different downstream gene regulation modelling studies.

[1] Duttke S.H. et al. ‘Position-dependent function of human sequence-specific transcription factors.’ Nature 2024;631:891–898.

[2] Rentzsch, P. et al. Nucleic acids research 2019:47(D1):D886–D894.

